# Low Water Absorption, High-Strength Polyamide 6 Composites Blended with Sustainable Bamboo-Based Biochar

**DOI:** 10.3390/nano10071367

**Published:** 2020-07-13

**Authors:** Shiliu Zhu, Yong Guo, Yuxia Chen, Shengquan Liu

**Affiliations:** Key Lab of State Forest and Grassland Administration on Wood Quality Improvement & High Efficient Utilization, Anhui Agricultural University, Hefei 230036, China; zhuslwood@163.com (S.Z.); sheherose@163.com (Y.C.)

**Keywords:** biochar, polyamide 6, water absorption, mechanical properties, composites

## Abstract

To promote the application of polyamide 6 (PA6) in wood–plastic composites, the negative effects associated with the thermal degradation of plant fibers must be overcome. In this study, waste bamboo fibers were subjected to pyrolysis and ball milling to afford nano bamboo-based biochar (BC), which was subsequently used as reinforcement to prepare PA6/BC nano composites by injection molding. In addition, the processing fluidity, water absorption, mechanical properties, and interface compatibility of PA6/BC composites were discussed. Results revealed that a BC content of less than 30 wt% is beneficial to improve the processing fluidity of the composites. With the increase in the BC content, the density of the PA6/BC composites gradually increased, while the water absorption of the PA6/BC composites gradually decreased, and the maximum decrease was 46%. Compared to that of pure PA6, the mechanical strength of PA6/BC composites was improved by the addition of BC, and the maximum tensile/flexural strength and modulus of PA6/BC composites increased by 41%/72% and 195%/244%, respectively. However, the impact strength decreased by 27%. After immersion treatment, the dimensional stability and mechanical strength of the composites decreased, while toughness improved. At a BC content of less than 40 wt%, BC particles exhibited good dispersibility and wettability in the PA6 matrix, and the rough surface and rich pore structure of BC rendered strong mechanical interlocking effects and good interface compatibility, thereby enhancing the mechanical properties of the composites.

## 1. Introduction

Wood–plastic composites (WPCs) are environmentally friendly and prepared by hot-pressing, extrusion, or injection molding, which is modified by blending agricultural and forestry processing residues (mainly plant fibers, such as wood/bamboo fiber, straw, and nutshell) with plastics [1,2,3]. WPCs exhibit dual advantages of plastics and plant fiber, and they are widely used in construction, vehicles, furniture, landscape, packaging, and other fields [4,5]. Certainly, WPCs still have the problems of low mechanical strength, high water absorption, and ductility, and researchers have improved these properties by adding surface-fibrillated plant fiber or cellulose nanofibers [6]. However, traditional WPCs are mostly based on general-purpose plastics with low mechanical properties (such as polyethylene, polypropylene, and polyvinyl chloride) [7] or degradable plastics (polylactic acid and poly(butylene succinate)) [8], while engineering plastics with high mechanical properties, such as polyamide and polycarbonate, are rarely used. Hence, the application of WPCs is limited in areas with high mechanical strength requirements, mainly because the melting point and processing temperature of engineering plastics are greater than 200 °C, and plant fibers gradually begin to degrade or gelatinize at this temperature [9,10], adversely affecting the composite. Therefore, inorganic fillers, such as glass fiber, mica, montmorillonite, gypsum powder, attapulgite, and talc [11,12,13,14], or carbon materials, such as carbon fiber, carbon black, carbon nanotubes, and graphene, are predominantly used for the blending modification of engineering plastics [15,16,17].

Polyamide 6 (PA6) is a semi-crystalline polymer with good heat resistance and low coefficient of friction. However, PA6 has high water absorption, resulting in a decrease in dimensional stability and mechanical strength [18]. Meanwhile, plant fibers also have greater water absorption and their processing temperature is not suitable for the modification of PA6. But the carbonization process can greatly reduce the water absorption of plant fibers and eliminate the constraints of processing temperature. Therefore, the modification of polyamide 6 with carbon-based materials is the most common method. For instance, Scaffaro et al. [19] have reported that plasma-modified carbon nanotubes significantly improve the mechanical, rheological, and electrical properties of PA6. Sun et al. [20] have reported that fluorinated graphite exhibits a heterogeneous nucleation ability in the PA6 matrix, thereby improving the crystallization performance of PA6 and promoting the formation of a stable α crystal form of PA6. As a result, the tensile strength and elastic modulus of the composites are considerably improved. These studies suggest that carbon-based materials play a significant role in the functional modification and performance improvement of PA6.

As a member of the carbon family, biochar has not been widely used for the modification of engineering plastics. Nevertheless, biochar also exhibits some excellent properties of carbon materials, such as strong electrical conductivity, good thermal conductivity, high specific surface area, and high hardness and modulus. Furthermore, among carbon-based materials, biochar is relatively less expensive as well as environmentally friendly. Studies have reported that biochar exhibits a strong interface interaction in a WPC system, which can improve the water resistance, bending properties, tensile properties, creep properties, electrical conductivity, and thermal properties of WPCs [21,22,23,24,25]. These studies have reported that biochar demonstrates immense potential, and it can replace carbon fiber as a filler for polymer modification, thereby reducing costs while simultaneously achieving high performance for composites [26,27].

Therefore, in this paper, bamboo-based biochar (BC) was used as a filler, and engineering plastic polyamide 6 (PA6) was used as the matrix to prepare PA6/BC composites by melt blending and injection molding. In addition, effects of the BC content on the processing fluidity, thermal performance, crystallization, dimensional stability, and mechanical properties of the composites were analyzed; the interface mechanism of BC particles and the PA6 matrix was revealed, and the optimal BC addition amount was determined so as to provide a preliminary reference for the in-depth study of PA6/BC composites.

## 2. Materials and Methods 

### 2.1. Materials

BC (average particle size of ~200 nm) was purchased from Shanghai Hainuo Charcoal Co., Ltd. (Shanghai, China) and carbonized in a tube furnace at a high temperature of 1100 °C (OTF-1200X, Hefei Kejing Materials Technology Co., Ltd., Hefei, China). Injection-grade polyamide 6 (PA6, YH3400), with a density of 1.157 g/cm^3^, was purchased from Chinapetrochemical Baling Co., Ltd. (Yueyang, China).

### 2.2. Methods

#### 2.2.1. Preparation of Bamboo Based Biochar Reinforced Polyamide 6 Composites

**Figure 1:**
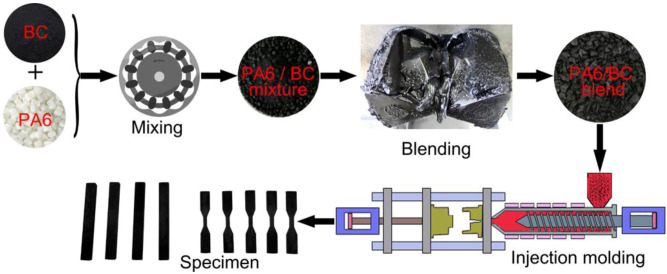
Processing diagram of polyamide 6/bamboo-based biochar (PA6/BC) composites.

Dry-processed BC and PA6 were evenly blended and placed in a torque rheometer (XSS-300, Shanghai Kechuang Rubber Plastics Machinery Set Co., Ltd., Shanghai, China) to completely mix and obtain a PA6/BC blend. The mixing temperature, mixing time, and rotation speed were 230 °C, 30 min, and 40 rpm, respectively. Next, the PA6/BC blend was added in a powerful plastic grinder (PC-400A, Longhe Plastic Machinery Co., Ltd., Chaozhou, China) and crushed into uniform particles, which were injected into a micro-injection molding machine (WZS10D, XINSHUO Precision Machinery Co., Ltd., Shanghai, China) to obtain a standard specimen for tensile strength, flexural strength, and impact strength tests. The barrel temperature of the injection molding machine was 265 °C, the mold temperature was 70 °C, the pressure was 0.6 MPa, and the holding time was 5 s. The specific preparation process is shown in Figure 1. The amount of the added BC was 10 wt%, 20 wt%, 30 wt%, 40 wt%, 50 wt%, and 60 wt% (mass fraction), and the corresponding composites were denoted as PA6/BC10, PA6/BC20, PA6/BC30, PA6/BC40, PA6/BC50, and PA6/BC60, while pure PA6 was used as a blank control.

#### 2.2.2. Characterization

##### Melt Flow Rate

The PA6/BC composite particles were placed in a melt flow rate meter (MFI-1211, Chengde Jinjian Testing Instrument Co., Ltd., Chengde, China) to measure the melt mass-flow rate (MFR). The experimental temperature was 265 °C, the nominal load was 2.16 kg, the reference time was 10 min, and the interval between cutting sections was 10 s. The MFR value was calculated according to ISO 1133-1-2011. The specific calculation method is shown in formula (1), where MFR (T, M) represents the melt mass-flow rate (g/10 min) at the test temperature T and M is the nominal load. T*_r_* is the reference time (600 s); m is the average mass of the cut (g); *t* is the time interval of the cut (s).

(1) $$\mathrm{MFR}\left(T,M\right)=T_{r}\times \frac{m}{t}$$

##### Water Absorption

The 24 h water absorption was determined according to our previous study [28]. Meanwhile, at room temperature (23 ± 1 °C), the PA6/BC specimen was immersed in distilled water for 30 days; the dimension and mass of the specimen were measured once a day, and the long-term water resistance and dimensional stability of the composite were analyzed. In addition, according to Fick’s second diffusion law, the diffusion coefficient of water in PA6/BC composites can be obtained from the linear part of the curve of the water absorption (*W_d_*) versus *d*^1/2^ (*d* represents the immersion days) [29,30], as shown in Equation (2). *W_0_*, *W_d_*, and *W_m_* represent the water content at *d* = *0*, *d*, and saturation equilibrium, respectively; *h* is the sample thickness; and *D* is the diffusion coefficient.

(2) $$\frac{W_{d}-W_{0}}{W_{m}-W_{0}}=\frac{4}{h}\times \sqrt{\frac{Dd}{\pi }}$$

To calculate the diffusion coefficient over a period of time, Equation (2) can be rewritten as Equation (3), where *W*_*d*1_ and *W*_*d*2_ represent the moisture content on immersion days *d*_1_ and *d*_2_, respectively. Therefore, $\frac{W_{d2}-W_{d1}}{\sqrt{d_{2}}-\sqrt{d_{1}}}$ is the slope (*k*) of the initial stage of the *W_d_* versus *d*^1/2^ curve; then, Formula (3) can be written as (4). The water absorption (*W_d_*) of the PA6/BC composite was plotted against *d*^1/2^, and the curve was fitted to obtain a slope *k*, which was subsequently used to calculate the diffusion coefficient *D* [31].

(3) $$D=\pi {\left(\frac{h}{4W_{m}}\right)}^{2}\times {\left(\frac{W_{d2}-W_{d1}}{\sqrt{d_{2}}-\sqrt{d_{1}}}\right)}^{2}$$

(4) $$D=\pi {\left(\frac{h}{4W_{m}}\right)}^{2}\times k^{2}$$

##### Mechanical Properties and Morphological Evaluation

Tensile strength and flexural strength tests were performed in accordance with ASTM D638-10 and ASTM D790-10, respectively, which were performed on a universal testing machine (AG-X Plux, Shimadzu, Kyoto, Japan). The total length of the tensile specimen was 63.5 mm, the width of the middle parallel section was 3.18 mm, the thickness was 3.54 mm, the gauge distance was 25 mm, and the loading speed was 10 mm/min. The size of the flexural specimen was 80 mm × 10 mm × 4 mm, the span was 60 mm, and the loading speed was 2 mm/min. The notched impact strength tests were performed in accordance with ASTM D256-10, and measured using a cantilever beam impact tester (XJUD, Shanghai Jiezhun Instrument Equipment Co., Ltd., Shanghai, China). The size of the specimen was 80 mm × 10 mm × 4 mm, and the notch depth was 2 mm. Meanwhile, the impact fracture surface was sprayed with gold and subjected to scanning electron microscopy (SEM, S-4800, Hitachi, Tokyo, Japan) analysis at an acceleration voltage of 3.0 kV. Five samples were used for each type of test, and the final result was based on its average value.

Under nitrogen protection, a three-point bending mode was utilized to perform dynamic thermomechanical analysis (DMA 242E, Netzsch, Selb, Germany) to obtain the glass transition temperature (T_g_), storage modulus (E’), loss modulus (E"), and loss factor (Tan δ) of the PA6/BC composites. The test temperature ranged from -50 to 210 °C, a heating rate of 3 °C/ min, a frequency of 1 Hz, an amplitude of 5 μm, a dynamic force of 3 N, a static force of 0.1 N, and a specimen size of 40 mm × 10 mm × 4 mm.

## 3. Results and Discussion

### 3.1. Melt Mass-Flow Rate (MFR)

**Figure 2:**
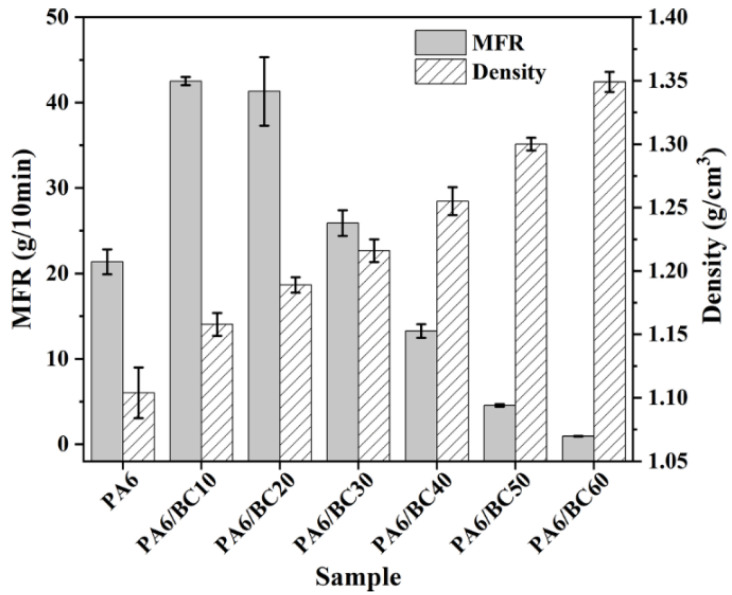
Melt mass-flow rate (MFR) and density of PA6/BC composites.

Figure 2 shows the MFR of PA6/BC composites with different BC contents. With the increase in the BC content, the MFR of PA6/BC composites first increased and then decreased. At a BC content of 10 wt% to 30 wt%, the MFRs of the PA6/BC composites were greater than that of pure PA6, the processing fluidity of the composites improved, and the maximum value (42.52 g/10 min) was achieved at a BC content of 10 wt%, which increased by 99.06% compared with that of pure PA6.

However, when the BC content was greater than 30 wt%, the MFR of the composite was less than that of pure PA6, and the processing fluidity worsened. Furthermore, the MFR of the composite reached the minimum value (0.94 g/10 min) at a BC content of 60 wt%, which was 95.60% less than that of pure PA6. This result is mainly related to the fact that the melt viscosity of pure PA6 is relatively large, and it easily adheres to the inner wall and die of the melt flow tester (a similar phenomenon also occurs in the barrel of the injection molding machine), affording high friction resistance and poor fluidity. By the addition of a BC content of 10 wt% to 30 wt%, a certain degree of lubrication was attained [32], reducing the frictional resistance of the barrel wall and die to the PA6 melt and improving the processing fluidity. However, at a BC content greater than 30 wt%, a high number of BC particles restricted the movement of the PA6 molecular chain, and the fluidity decreased. Therefore, a BC content of up to 30 wt% is recommended to ensure good processing fluidity for the PA6/BC composites. In addition, Figure 2 also shows that with the increase in the BC content, the density of the PA6/BC composites gradually increased, from 1.104 g/cm^3^ (pure PA6) to 1.349 g/cm^3^ (PA6/BC60), corresponding to a 22.19% increase.

### 3.2. Water Absorption

**Figure 3:**
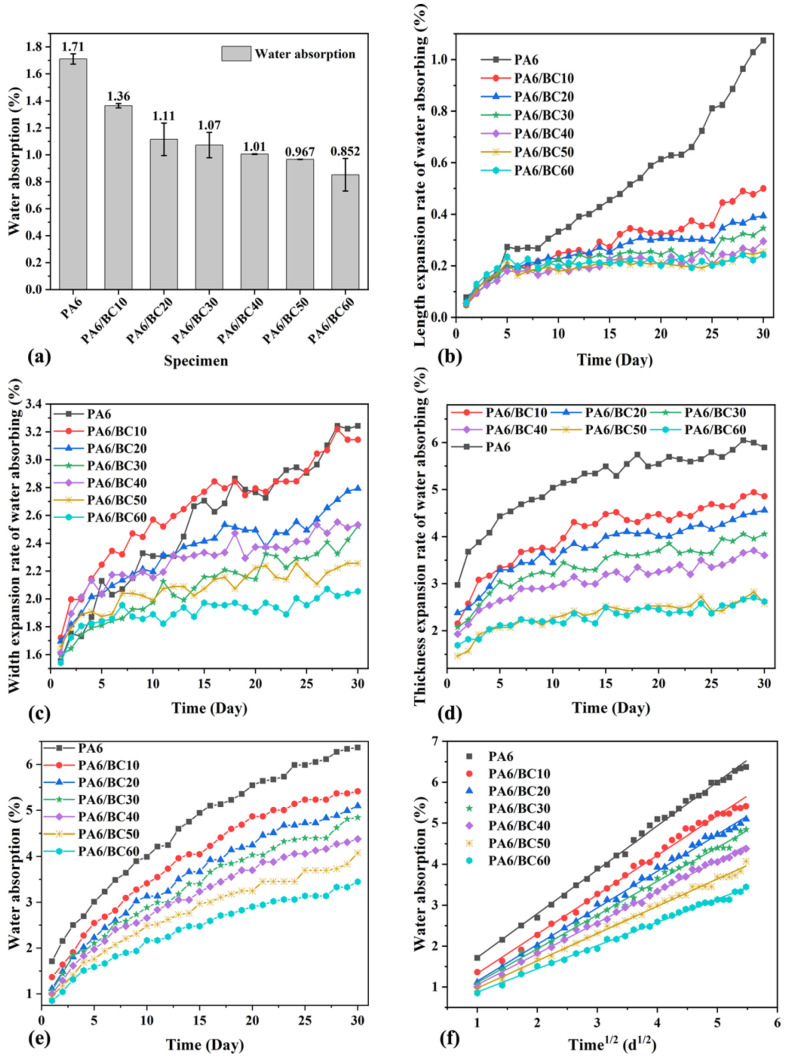
(**a**) The 24 h water absorption rate of the PA6/BC composites; (**b**–**d**) length/width/thickness expansion of water absorption for PA6/BC composites within 30 days; (**e**) water absorption of PA6/BC composites within 30 days; and (**f**) fitting curves based on Fick’s second law.

Figure 3 shows changes in the water absorption and dimensional expansion of PA6/BC composites after immersion treatment. The addition of BC led to the reduction in the 24 h water absorption and improvement in the water resistance of the PA6/BC composites (Figure 3a). The addition of 60 wt% BC led to a minimum water absorption rate of 0.852%; this value is 50.18% less than that of pure PA6, mainly because the PA6 molecular chain exhibits strong polarity due to the presence of amide groups (–NHCO–), which exhibit strong hydrophilicity [18]. However, after the carbonization of BC at 1100 °C, surface oxygen-containing functional groups and hydrophilic groups were eliminated, and the aromaticity was strong, leading to enhanced hydrophobicity [33,34,35]. Therefore, by the addition of hydrophobic BC into the PA6 matrix as a filler, the content of hydrophilic PA6 decreased, while the content of hydrophobic BC gradually increased, leading to the decrease in the water absorption of the composite. In addition, with the increase in the BC content, the change in the water absorption rate gradually became smaller; this result is mainly related to the fact that as the BC content is extremely high, the coating between PA6 and BC worsened, there are some gaps or holes in the interface, and the BC surface exhibits a rich pore structure; in addition, some of the pores also store water, weakening the impact of the reduced PA6 content.

Figure 3b–e revealed that with the increase in the immersion treatment time, the length/width/thickness expansion rate and water absorption of the PA6/BC composites gradually increased. However, the water absorption of the PA6/BC composites exhibited anisotropy, and the expansion rate in the thickness direction was clearly greater than that in the width and length directions. On the 30th day, the length, width, and thickness expansion rates and water absorption of pure PA6 reached 1.07%, 3.24%, 5.89%, and 6.37%, respectively, while the corresponding values for PA6/BC composites gradually decreased with the increase in the BC content and reached the minimum value at a BC content of 60 wt%, corresponding to 0.24%, 2.05%, 2.62%, and 3.45%, respectively. Compared with those of pure PA6, the length/width/thickness expansion rate and water absorption of the PA6/BC60 decreased by 77.57%, 36.73%, 55.52%, and 45.84%, respectively. This result further indicated that the addition of BC leads to the improved water resistance of the PA6/BC composites, reduces the impact of the water on the dimensional stability, and exhibits better applicability in the water environment than pure PA6.

Combined with Fick’s second diffusion law, the water absorption kinetics of PA6/BC composites was analyzed, and the diffusion coefficient of water in PA6/BC composites was calculated. Figure 3f shows the linear fitting curve of the water absorption to *d*^1/2^, and Table 1 shows the fitting curve equation, the correlation coefficient (R^2^), the water absorption of the PA6/BC composite on the 30th day (*W_30_*/%), and the diffusion coefficient (*D*). Although the high R^2^ was due to the same sample being tested over time which violates the assumption of independence between data points and inflates the R^2^. However, Figure 3f and the R^2^ value also suggested that the water absorption and *d*^1/2^ exhibit an approximately linear increase, indicating that the water absorption process of the PA6/BC composite satisfies Fick’s second diffusion law [31]. In addition, The *D* values for PA6/BC10, PA6/BC20, PA6/BC30, and PA6/BC40 were 12.17%, 9.06%, 3.02%, and 6.04%, respectively, greater than those of pure PA6, while the *D* values for PA6/BC50 and PA6/BC60 were 4.97% and 3.99%, respectively, less than those of pure PA6, respectively. At a low BC content, the polar amide groups on the PA6 molecular chain still dominated the water absorption process, and the pores on BC and the interface gap between BC and PA6 also became the main direction for water diffusion, leading to a more rapid rate of water diffusion. At a high BC content, the PA6 content considerably reduced, the attraction of polar amide bonds to water molecules was relatively reduced, the hydrophobic effect of BC was gradually reflected, and the water diffusion coefficient was slightly reduced. However, owing to the rich pore structure of BC and the presence of amide groups on the PA6 molecular chain, the diffusion of water in the PA6/BC composites continuously occurs. It is difficult for the water absorption of the PA6/BC composites to attain a long-term saturation equilibrium state. Only the short-term relative saturation equilibrium shown in Figure 3f can be reached. With the infinite extension of the immersion time, water degradation of PA6 occurred, the interface between PA6 and BC was destroyed, the interface gap increased, this saturation state was broken, and the water absorption increased slowly again.

### 3.3. Mechanical Properties

**Figure 4:**
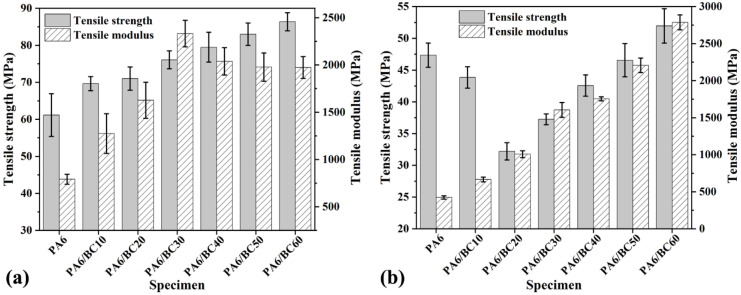
Tensile properties of PA6/BC composites with different BC contents, (**a**) no water treatment, (**b**) water treatment.

Figure 4a,b shows the tensile strength and tensile modulus of PA6/BC composites with different BC contents before and after water immersion treatment, respectively. From Figure 4a, BC particles exhibited a strong enhancement effect on PA6, and the tensile strength and tensile modulus of PA6/BC composites were greater than those of pure PA6. With the increase in the BC content, the tensile strength of PA6/BC composites gradually increased, and the tensile modulus first increased and then decreased. The tensile strength and tensile modulus of pure PA6 were 61.14 MPa and 790.89 MPa, respectively. At a BC content of 60 wt%, the tensile strength of PA6/BC60 reached the maximum of 86.36 MPa, corresponding to an increase of 41.25% compared with that of pure PA6. When the BC content was 30 wt%, the tensile modulus of PA6/BC30 reached the maximum of 2331.73 MPa, corresponding to an increase of 194.82% compared with that of pure PA6. This result indicated that the PA6/BC system exhibits good interfacial compatibility and a strong stress-transfer ability at the interface. BC with high hardness and modulus can bear the applied stress better in a composite system, effectively strengthening the PA6 matrix. After immersion treatment of 30 days, the tensile strength of PA6/BC composites was apparently reduced (Figure 4b) mainly because of the presence of hydrophilic polar amide groups in the PA6 matrix. Water immersion treatment caused partial hydrolysis. Meanwhile, after water treatment, the flexibility of the PA6 molecular chain was enhanced, but the strength was decreased. Furthermore, water immersion treatment destroyed the interface of PA6/BC composites, weakened the interface interaction, and reduced the interface strength, leading to the decrease in the overall strength of the composites. Overall, after immersion treatment of 30 days, the tensile modulus of PA6/BC composites was greater than that of pure PA6. The tensile strength of only PA6/BC60 was 9.74% greater than that of pure PA6.

**Figure 5:**
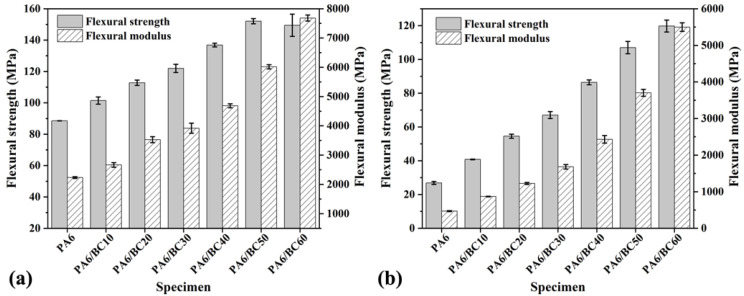
Flexural properties of PA6/BC composites with different BC contents, (**a**) no water treatment, (**b**) water treatment.

Figure 5a,b shows the flexural strength and modulus of PA6/BC composites before and after water immersion treatment, respectively. The addition of BC led to the significant improvement in the flexural strength and flexural modulus of PA6, exhibiting a strong reinforcement effect. The flexural strength and flexural modulus of pure PA6 were 88.55 MPa and 2234.32 MPa, respectively. With the increase in the BC content, the flexural strength of PA6/BC composites first increased and then decreased, while the flexural modulus gradually increased. By the addition of a BC content of 50 wt%, the flexural strength of PA6/BC50 reached the maximum value of 152.04 MPa; this value is 71.70% greater than that of pure PA6, and the flexural modulus also increased by 169.29%. Moreover, the flexural modulus reached the maximum value (7680.45 MPa) at a BC content of 60 wt%; this value is 243.75% greater than that of pure PA6. In the treatment of BC at 1100 °C, it exhibited high porosity as well as a high specific surface area, thereby rendering a strong capillary effect, improving the infiltration between BC and PA6, and forming a large number of van der Waals bonds with the PA6 molecular chain; all these factors in turn lead to a good interface interaction. Simultaneously, the higher surface roughness of BC also enhanced its adhesion and mechanical interlocking with PA6 [36], thereby improving the bonding strength and deformation resistance of the composite interface. However, at a BC content of greater than 50 wt%, the coating of PA6 on BC was weakened, and the dispersibility of BC worsened, leading to stress concentration and fragile points due to agglomeration and failure under a low load. In addition, high-temperature carbonized BC exhibited higher hardness and modulus [37], which in turn can increase the hardness and modulus of the PA6 matrix. Moreover, BC in the PA6 matrix can limit the movement of the PA6 molecular chain and also increase its rigidity. Therefore, the flexural modulus of the PA6/BC composite increases with the increase in the BC content. After immersion treatment of 30 days, the flexural strength and flexural modulus of PA6/BC composites significantly decreased, and there are similar results in traditional WPCs [38]. However, with the increase in the BC content, the flexural strength and flexural modulus of PA6/BC composites gradually increased. The flexural strength and flexural modulus of pure PA6 after immersion treatment were 26.85 MPa and 469.31 MPa, which were 69.68% and 78.99% less than those before immersion treatment. For PA6/BC60, the maximum flexural strength and flexural modulus were 119.76 MPa and 5498.01 MPa, which decreased by 19.87% and 28.42%, respectively. This result indicated that the addition of BC helps to maintain the remaining strength of the PA6/BC composite after immersion treatment.

**Figure 6:**
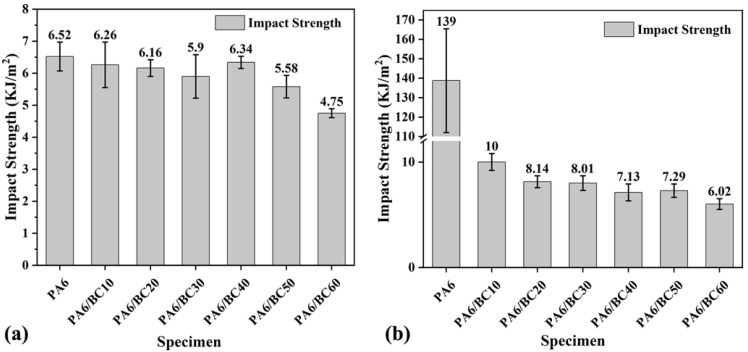
Impact strength of PA6/BC composites with different BC contents, (**a**) no water treatment, (**b**) water treatment.

Figure 6a,b show the notched impact strength of PA6/BC composites before and after water immersion treatment, respectively. The addition of BC particles led to the reduction of the notched impact strength of PA6, and with the increase in the BC content, the downward trend became more clear. At a BC content of 60 wt%, the notched impact strength of PA6/BC60 reached the minimum value of 4.75 KJ/m^2^; this value is 27.15% less than that of pure PA6, mainly because high-temperature carbonized BC particles exhibit high rigidity, and the addition to PA6 can effectively improve the rigidity of the composites; however, the presence of BC particles also limited the mobility of the PA6 molecular chain and decreased its flexibility. The plastic deformation of the composites was constrained, and the energy dissipation was reduced. Then, the ability of the composites to resist the damage of impact load weakened, and the toughness decreased. Notably, with the increase in the BC content, the PA6 molecular chain became increasingly constrained, and its ability to resist impact load further declined, leading to the sharp decrease in toughness. Interestingly, the notched impact strength of PA6/BC40 was greater than that of the other PA6/BC composites, probably because PA6/BC40 exhibited the best interface compatibility under this ratio, and the interfacial bonding strength was high. A good interface effectively dissipated a part of the impact energy and improved the impact toughness of the composite. After immersion treatment of 30 days, the toughness of PA6/BC composites was improved, and the notched impact strength increased. After immersion treatment, the PA6 molecular chain absorbed a considerable amount of water, and the flexibility of the chain was enhanced. Furthermore, the erosion of water molecules destroyed the interface of PA6/BC, the restriction of BC particles on the PA6 molecular chain was weakened, and the mobility of the PA6 molecular chain was enhanced. Then, the ability of the composites to withstand plastic deformation was enhanced, and high impact energy dissipated. After immersion treatment, the notched impact strength of pure PA6 was increased by 20.32 times, exhibiting a clear toughening phenomenon in water, while the notched impact strength values for PA6/BC10, PA6/BC20, PA6/BC30, PA6/BC40, PA6/BC50, and PA6/BC60 increased by 59.74%, 32.14%, 35.76%, 12.46%, 30.65%, and 26.74%, respectively. PA6/BC40 exhibited the smallest increase, further indicating that PA6/BC40 exhibits better interface bonding and that water molecules exhibit less erosion and damage to the interface of the composites. The BC particles still exhibited a strong limiting effect on the PA6 molecular chain.

### 3.4. Dynamic Thermomechanical Analysis (DMA)

**Figure 7:**
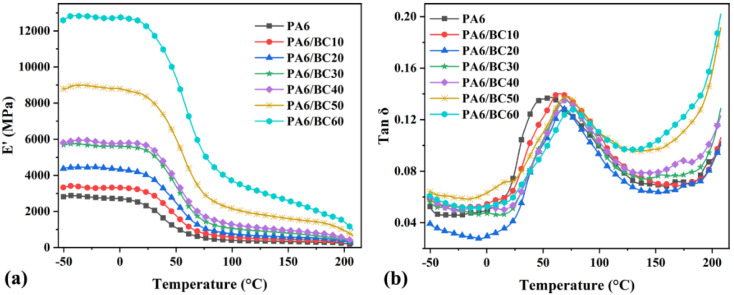
Dynamic thermomechanical analysis (DMA) curves of PA6/BC composites with different BC contents, (**a**) E’, (**b**) tan δ.

Figure 7a,b shows the relationship between storage modulus (E’), loss factor (tan δ), and temperature of PA6/BC composites with different BC contents. Table 2 summarizes the main characteristic parameters from the DMA curves, including the maximum storage modulus (E’_max_), the on-point temperature of the storage modulus step-down (T_o_), the peak loss modulus (E”_p_) and its corresponding temperature (T_E”p_), and the loss factor peak (Tan δ_p_) and its corresponding temperature (glass transition temperature, T_g_). The maximum storage modulus of pure PA6 was 2882.00 MPa. Then, with the increase in the BC content, the storage modulus of PA6/BC composites gradually increased and reached its maximum value (12844.00 MPa) at a BC content of 60 wt%; this value is 345.66% greater than that of pure PA6. This result indicated that the addition of BC leads to remarkable improvement in the rigidity of the PA6/BC composite. The higher the BC content, the more clear the improvement effect, mainly because high-temperature carbonized BC particles exhibit higher hardness and rigidity [37]. A large number of BC particles form a rich interface structure in the PA6 matrix via van der Waals bonds and mechanical adhesion, and the applied stress can be transferred to the high modulus BC particles, thereby increasing the storage modulus of the composites. Meanwhile, the loss modulus of the composites also gradually increased with the increase in the BC content. E”_p_ increased from 220.00 MPa for pure PA6 to 892.00 MPa for PA6/BC60, which increased by 305.45%. Owing to the good mechanical adhesion between the PA6 molecular chain and BC particles, the mobility and relaxation of the PA6 molecular chain were restricted by the BC particles, and the restriction was stronger at a high BC content. Under external force, the BC particles and PA6 matrix rubbed against each other through the interface to consume energy, thereby increasing the energy consumption during the movement of the PA6 molecular chain as well as the loss modulus of the composites [39]. However, from the perspective of the loss factor, it slightly fluctuated with the increase in the BC content, indicating that with the increase in the BC content, the growth rates of loss modulus and storage modulus are not consistent; hence, the ratio (tan δ) fluctuates.

In addition, as can be observed from Table 2, with the increase in the BC content, T_o_, T_E”p_, and T_g_ gradually increased. The increased T_o_ indicated that the temperature required for the thermal movement of the PA6 molecular chain is increased and that the thermal stability of the PA6/BC composites is improved. Similarly, the increase in T_E”p_ indicated that the energy required for the maximum internal friction of the composites increases with the increase in the BC content. The continuous increase in temperature is required to meet the thermal movement requirements of highly filled PA6/BC composites. The glass transition temperature (T_g_) reflected the movement of the PA6 molecular chain in the amorphous region. Pure PA6 did not exhibit compatibility problems, and the molecular chain motion was not hindered; hence, T_g_ is observed at a low temperature. However, in the PA6/BC composite system, the BC particles restricted the movement of the PA6 molecular chain in the amorphous region, which required a high energy and free volume to complete the glass transition, and this limitation increased with the increase in the BC content. Therefore, T_g_ increases with the increase in the BC content.

### 3.5. Scanning Electron Microscopy (SEM)

**Figure 8:**
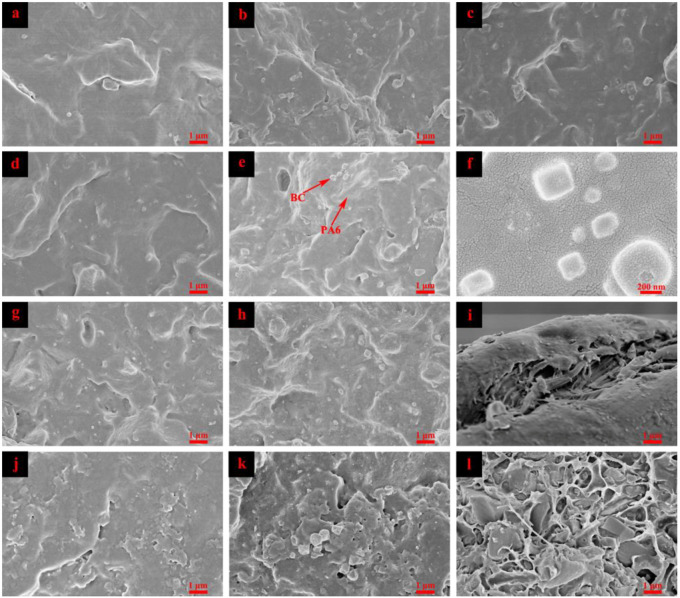
Scanning electron microscope (SEM) images of the impact section of PA6/BC composites with different BC contents, (**a**,**i**) PA6; (**b**,**j**) PA6/BC10; (**c**) PA6/BC20; (**d**) PA6/BC30; (**e**,**f**,**k**) PA6/BC40; (**g**) PA6/BC50; (**h**,**l**) PA6/BC60 (**i–l** represent the sample treated by water immersion).

Figure 8 shows the SEM images of the impact fracture surface of pure PA6 and PA6/BC composites. PA6 exhibited a relatively flat fracture surface (Figure 8a). However, after the addition of BC particles, the surface roughness increased, as well as undulations and local protrusions, mainly because, by the action of external force, the PA6 matrix of the composites was deformed, but it was bound and pulled by the BC particles, and the stress was transferred from the PA6 matrix to the BC particles; hence, an increased number of undulating surfaces are observed during breakage. This result also indicated that the PA6 matrix and BC particles exhibit a good interface compatibility, where stress can be effectively transmitted, and the enhancement effect of BC particles is achieved. In addition, the number of BC particles on the fracture surface was confirmed to be small, the dispersibility was uniform, and few pores were generated by particle debonding. The PA6 matrix effectively coated the BC particles, and the phase separation was not clear. The BC particles adhered well to the PA6 matrix and formed a tighter binary structure, which was beneficial to the improvement of the mechanical properties of the composites. However, with the change in the BC content, the interface of PA6/BC composites also apparently changed. At a low BC content, the scattered BC particles formed an obvious “island structure” on the PA6 matrix (Figure 8b–d). Then, with the gradual increase in the BC content, the BC particles scattered on the PA6 matrix gradually became dense, and even agglomeration and debonding in local areas were observed, resulting in some holes (Figure 8e–h). However, SEM magnification images revealed that the BC particles and PA6 matrix still exhibit good adhesion, with no clear separation, and interfacial gaps are not observed. In addition, a layer of white material was adhered onto the BC particles, probably because BC particles played a heterogeneous nucleation role in the composite system, and PA6 molecular chains stacked and formed along the BC surface to form tiny crystal regions. Precisely because of the good interface combination of the PA6 matrix and BC particles, PA6/BC composites can exhibit excellent mechanical strength. However, BC particles with high hardness and modulus inevitably weakened the toughness of the PA6 matrix while strengthening it. In the experiment, small-sized BC particles were used, with a high specific surface area and surface energy and good wettability, and these particles were embedded in the PA6 matrix, generating a large number of van der Waals bonds and forming a relatively stable binary structure. In addition, BC particles with a rich pore structure exhibited a strong capillary effect, and the diffusion effect of the PA6 molecular chain was enhanced, whereby it can flow into the pores of BC to form a more stable mechanical interlocking structure [40]. The stress-bearing and transmission capacity of the composite system improved with good interface combination, and the mechanical properties were enhanced.

SEM images (Figure 8i–l) of the fracture surface of pure PA6 and PA6/BC composites after immersion treatment of 30 days revealed a rougher surface. The fracture surface of pure PA6 was no longer smooth and flat, and layers of cracks were observed. This result revealed that after water immersion treatment, PA6 exhibits ductile fracture and enhanced toughness; this result is consistent with the results obtained from impact strength tests. In the PA6/BC system, the holes generated by the debonding of BC particles increased, and the interface of the composite system was severely damaged by water molecules. Moreover, the BC particles and PA6 matrix exhibited clear interface gaps, the interface interaction was weakened, which in turn reduced the interface bonding strength and weakened the stress transmission effect; this in turn led to decreased mechanical strength. This result indicated that the improvement in the interfacial compatibility between the PA6 matrix and BC particles is the key to completely exert the function of BC reinforcement. In this experiment, the PA6 matrix can effectively coat the BC particles to form a compact heterogeneous binary structure, thereby considerably improving the composite strength. However, SEM analysis revealed that BC particles agglomerate in a local area in the high-fill system; hence, the amount of added BC is preferably controlled within 40% to maintain its good comprehensive performance.

## 4. Conclusions

In this study, PA6/BC composites with different contents of added BC were prepared by melt blending and injection molding. Effects of the BC content on the interface regulation and performance of PA6/BC composites were discussed from the aspects of the melt flow rate, water absorption, mechanical properties, and interface compatibility. The enhancement mechanism and interface mechanism of BC were analyzed. Considering the strength, toughness, processing fluidity, and dispersion effect of BC particles in the PA6/BC system, it is believed that when the amount of BC added is 30 wt% to 40 wt%, the PA6/BC composites exhibited the best comprehensive performance. At this point, the better mechanical strength, dimensional stability, and water resistance of the PA6/BC composites can be maintained, and a good interfacial compatibility between PA6 matrix and BC particles can be ensured, thereby maintaining the toughness and processing fluidity of the composites to the highest extent.

Compared to traditional WPC and pure PA6, the PA6/BC composites are more cost-effective, and realize the high value-added utilization of agricultural and forestry wastes. Moreover, PA6/BC composites exhibit excellent physical and mechanical properties, and have great application potential for the fields of furniture, construction, packaging, automobiles, aerospace, and bridges.

## Figures and Tables

**Table 1:** Parameters of water absorption kinetics for fitting curves based on Fick’s second law.

Specimen	Fitting Curve Equation	R^2^ value	*W_30_*/%	*D*/ × 10^−3^ mm^2^·s^−1^
PA6	W = 0.65057 + 1.07107 t^1/2^	0.99674	6.37	10.27
PA6/BC10	W = 0.36733 + 0.96303 t^1/2^	0.99344	5.41	11.52
PA6/BC20	W = 0.24816 + 0.89537 t^1/2^	0.99653	5.10	11.2
PA6/BC30	W = 0.28166 + 0.82569 t^1/2^	0.99517	4.84	10.58
PA6/BC40	W = 0.29742 + 0.75818 t^1/2^	0.99708	4.38	10.89
PA6/BC50	W = 0.30602 + 0.66704 t^1/2^	0.9953	4.07	9.76
PA6/BC60	W = 0.30806 + 0.56831 t^1/2^	0.99569	3.45	9.86

**Table 2:** Characteristic data for dynamic thermomechanical analysis of PA6/BC composites with different BC contents.

Specimen	E’_max_ (MPa)	T_o_ (°C)	E"_p_ (MPa)	T_E"p_ (°C)	T_g_ (°C)	Tan δ_p_
PA6	2882.00	21.20	220.00	31.80	56.90	0.137
PA6/BC10	3414.00	24.10	253.00	36.50	64.40	0.140
PA6/BC20	4462.00	28.20	268.00	41.70	68.40	0.128
PA6/BC30	5751.00	30.90	377.00	42.90	69.50	0.135
PA6/BC40	5954.00	31.50	394.00	42.80	69.70	0.135
PA6/BC50	8998.00	36.50	693.00	43.10	71.50	0.138
PA6/BC60	12,844.00	37.90	892.00	46.40	75.90	0.128

E’_max_: maximum storage modulus, T_o_: on-point temperature of the storage modulus step-down, E"_p_: peak loss modulus, T_E"p_: temperature at the peak of loss modulus, Tan δ_p_: peak loss factor, and T_g_: glass transition temperature.
